# A Pharmacogenetic Study of *CYP2C19* in Acute Coronary Syndrome Patients of Colombian Origin Reveals New Polymorphisms Potentially Related to Clopidogrel Therapy

**DOI:** 10.3390/jpm11050400

**Published:** 2021-05-12

**Authors:** Mariana Angulo-Aguado, Karen Panche, Caroll Andrea Tamayo-Agudelo, Daniel-Armando Ruiz-Torres, Santiago Sambracos-Parrado, Maria Jose Niño-Orrego, Nathaly Páez, Laura B Piñeros-Hernandez, Luisa-Fernanda Castillo-León, Juan Mauricio Pardo-Oviedo, Katherine Parra Abaunza, Paul Laissue, Nora Contreras, Carlos Alberto Calderón-Ospina, Dora Janeth Fonseca-Mendoza

**Affiliations:** 1 Center for Research in Genetics and Genomics—CIGGUR, GENIUROS Research Group, School of Medicine and Health Sciences, Universidad Del Rosario, Carrera 24 N° 63C-69, 112111 Bogotá, Colombia; mariana.anguloa@urosario.edu.co (M.A.-A.); caroll.tamayo@urosario.edu.co (C.A.T.-A.); danielar.ruiz@urosario.edu.co (D.-A.R.-T.); santiago.sambracosp@urosario.edu.co (S.S.-P.); mariaj.nino@urosario.edu.co (M.J.N.-O.); Nathalya.paez@urosario.edu.co (N.P.); laurab.pineros@urosario.edu.co (L.B.P.-H.); lu-casti@hotmail.com (L.-F.C.-L.); paullaissue@yahoo.com (P.L.); nora.contreras@urosario.edu.co (N.C.); carlos.calderon@urosario.edu.co (C.A.C.-O.); 2 Internal Medicine Department, School of Medicine and Health Sciences, Hospital Universitario Mayor-Méderi, Universidad del Rosario, Carrera 24 N° 63C-69, 112111 Bogotá, Colombia; karen.panche@urosario.edu.co (K.P.); juan.pardo@urosario.edu.co (J.M.P.-O.); katherinea.parra@urosario.edu.co (K.P.A.); 3 Biopas Laboratoires, Orphan Diseases Unit, BIOPAS GROUP, 111111 Bogotá, Colombia

**Keywords:** platelet reactivity, single-nucleotide variants, pharmacogenetics, acute coronary syndrome, clopidogrel, genotype, allele, polymorphism

## Abstract

Clopidogrel, an oral platelet P2Y_12_ receptor blocker, is used in the treatment of acute coronary syndrome. Interindividual variability in treatment response and the occurrence of adverse effects has been attributed to genetic variants in *CYP2C19*. The analysis of relevant pharmacogenes in ethnically heterogeneous and poorly studied populations contributes to the implementation of personalized medicine. We analyzed the coding and regulatory regions of *CYP2C19* in 166 patients with acute coronary syndrome (ACS) treated with clopidogrel. The allele frequencies of *CYP2C19 alleles* *1, *2, *4, *17, *27 and *33 alleles were 86.1%, 7.2%, 0.3%, 10.2%, 0.3% and 0.3%, respectively. A new potentially pathogenic mutation (p.L15H) and five intronic variants with potential splicing effects were detected. In 14.4% of the patients, a new haplotype in strong linkage disequilibrium was identified. The clinical outcome indicated that 13.5% of the patients presented adverse drugs reactions with a predominance of bleeding while 25% of these patients were carriers of at least one polymorphic allele. We propose that new regulatory single-nucleotide variants (SNVs) might potentially influence the response to clopidogrel in Colombian individuals.

## 1. Introduction

Dual antiplatelet therapy with clopidogrel and aspirin has been routinely recommended in the American College of Cardiology/American Heart Association guidelines for patients with acute coronary syndrome to prevent atherothrombotic events [1,2]. Clopidogrel is an inactive prodrug that is converted into an active metabolite by two-step biotransformation. This metabolite binds irreversibly to the purinergic adenosine diphosphate (ADP) platelet receptor P2Y_12_ and inhibits ADP-stimulated platelet aggregation [3,4]. Clopidogrel is one of the most widely used platelet antiaggregant with over 40,000,000 patients prescribed worldwide [5,6]. Although clopidogrel has high clinical efficacy, over 30% patients exhibit significant interindividual variability in platelet inhibition, which reduces the antithrombotic effect of the medication [7]. Inadequate response to clopidogrel is associated with a higher risk of cardiac events and bleeding in patients that show high platelet reactivity (HPR) or low platelet reactivity (LPR), respectively [4,8]. Interindividual variability in the response to clopidogrel is influenced by clinical and environmental factors such as age, diabetes, body mass index, triglycerides and drug–drug interactions [9,10]. In addition to these clinical factors, genetic polymorphisms implicated in the pharmacokinetics and pharmacodynamics of clopidogrel are considered determinants in the response to anti-aggregation therapy, and heritability appears to be responsible for over 70% of interindividual variability [6,7,11]. To date, association studies between genetic variants, cardiovascular events, HPR and LPR have focused on polymorphisms on *PON1 (p.Q192R), ABCB1 (C.3435C>T)* polymorphisms, and particularly the *CYP2C19* gene [12,13,14,15]. CYP2C19, an enzyme of the cytochrome P450 (CYP450) superfamily, is considered the key enzyme related to the bioactivation of clopidogrel through the two-step oxidative process that leads to the formation of 2-oxo-clopidogrel and the active metabolite clopi-H4 [16,17]. Only some genetic variants of *CYP2C19* have been widely explored and their relation to the therapeutic response to clopidogrel has been established. The CYP2C19*2 (c.681 G>A, rs4244285) and *3 (c.636 G>A, rs4986893) alleles are considered loss-of-function (LOF) alleles and have been associated with higher platelet aggregation induced by ADP and a higher risk of atherothrombotic events [18,19]. The CYP2C19*17 (c.−806C>T, rs 12248560), a gain-of-function allele has been associated with ultrarapid metabolism that leads to the increase of platelet inhibition and a higher risk of bleeding. Response to clopidogrel can be assessed by determining the platelet function through the quantification of platelet reactivity. Diverse methods such as ADP-induced light transmittance aggregometry (LTA), the Verify Now P2Y_12_ assay and the INNOVANCE PFA-200 system, determine the potential anti-aggregation effectiveness of the drug. Few reports correlating molecular genotype and platelet reactivity, a potential positive association between these two factors has been determined [20]. Variations in gene allele frequencies are common across populations and can contribute to differences in the treatment effectiveness, which impacts the prevalence of HPR due to LOF *CYP2C19* alleles [17,21].

There have been many studies about *CYP2C19* on individuals with predominantly European ancestry, which limits the clinical implementation of pharmacogenetics in understudied populations, such as Latin Americans. 

Due to the key role of loss and gain-of-function *CYP2C19* alleles in therapeutic response to clopidogrel, new studies for Latin American subgroups are necessary to identify susceptibility polymorphisms and their association to the response to clopidogrel. 

In the present study, in order to assess the potential association of *CYP2C19* polymorphic alleles with platelet reactivity in acute coronary syndrome (ACS) patients, we studied the promoter, coding regions and intron-exon boundaries of the gene. Our results indicated that 33.7% of ACS patients were carriers of at least 1 polymorphic allele in *CYP2C19,* 7.8% of which were loss-of-function variants and 10.2% gain-of-function alleles. Our study identified intronic variants with potential splicing alterations leading to the generation of new predicted cryptic splicing sites or branch point modifications. 

To our knowledge, this study is the first analysis of the *CYP2C19* gene and platelet reactivity assessment using the INNOVANCE PFA-200 system in a Latin American Population. These results reveal new polymorphisms worth considering in the implication of pharmacogenetics-based clopidogrel therapy in the Colombian population.

## 2. Patients and Methods

### 2.1. Sampling and Data Collection

This study included 166 patients who received care in the Hospital Universitario Mayor-Méderi, located in Bogota (Colombia). Eligible patients were invited to participate in the study. Those who accepted signed informed consent. The study included patients 18 years or older admitted to the hospital due to acute coronary syndrome who received a dose of clopidogrel of 300 mg and then 75 mg dose for at least seven consecutive days. The clinical management of clopidogrel therapy and the dose indicated for the patients were performed according to the guidelines specified in the national clinical practice guide for acute coronary syndrome (http://gpc.minsalud.gov.co/ (accessed on 14 March 2021)). All patients included in the study received treatment with clopidogrel for at least 7 days. However, according to medical criteria, 13 of them subsequently changed their antiplatelet therapy (12 to ticagrelor and 1 to prasugrel).

The study excluded individuals who were using oral anticoagulants and glycoprotein IIb/IIa receptor inhibitors, with hematocrit values <25% or >52%, platelet count <100 × 10^9^/L, creatinine >15 mg/dL, clinical evidence of liver damage or profound alteration of platelet function.

Variables in patients were compiled; these variables included information such as demographics characteristics, gender, type of acute coronary syndrome (unstable angina, acute infarction with or without ST elevation) and comorbidities (e.g., diabetes, hypertension and obesity). 

All experimental procedures were approved by the Ethics Committee of Universidad del Rosario. The study followed the guidelines of the Declaration of Helsinki. (Approved DVO005 990-CV1018, institutional review board reference IV-FPC015 and ABN062).

### 2.2. Genotyping of the Promoter and Coding Regions of CYP2C19

Genomic DNA was obtained from blood samples of 166 patients using the Quick-DNA™ Miniprep Plus Kit (Zymo research). Using PCR, the coding region of *CYP2C19* (9 exons), the intron-exon boundaries, and the promoter (−1 to −1500 base pairs relative to transcriptional start site) were amplified. PCR products were purified and sequenced directly using Sanger sequencing. Primers were designed using Primer3 (Supplementary Table S1). The reference sequence was obtained from the Ensembl database (ENST00000371321.9).

To define the *CYP2C19* alleles, we used the information described in The Human CYP allele nomenclature database (http://www.cypalleles.ki.se/ (accessed on 14 March 2021)). Variants were considered novel when they were not previously reported in public databases or literature. We used the PolyPhen2, SIFT, and MutPred software to predict the effect of the amino acid substitution on protein function. Sequences of proteins with modified residues were compared with orthologous proteins of mammalian species using available public database sequences (https://www.uniprot.org/uniprot/ (accessed on 14 March 2021)), to assess evolutionary conservation. For the splice variants, we realized an in silico prediction using Human Splicing Finder v3.1 (http://www.umd.be/HSF/ (accessed on 14 March 2021)) and Alamut software (v2.15) (http://downloads.interactive-biosoftware.com (accessed on 14 March 2021)). Linkage disequilibrium was assessed for variants c.681G>A (rs 4244285), c.819 + 228 A>G (rs12571421) and c. 332-23A>G (rs12769205) using Haploview 4.2 (https://www.broadinstitute.org/ (accessed on 14 March 2021)).

Promoter variants were identified using Ensembl database ENST00000371321.9 (https//www.ensembl.org/ondex.html (accessed on 14 March 2021)). In the cases where the promoter region or intronic variant defined a specific allele, it was assigned according to the information in The Human CYP Allele Nomenclature database (http://www.cypalleles.ki.se/ (accessed on 14 March 2021))

### 2.3. Platelet Function Test

The platelet function was assessed using the INNOVANCE PFA-200 P2Y system (Siemens Healthcare). Blood samples were obtained at least 4 h after the administration of the loading dose of clopidogrel. The samples were obtained in tubes containing 3.2% sodium citrate. The PFA-200 P2Y is a system used for the assessment of platelet function by simulating the process of primary hemostasis in vitro. The assay simulates in vitro the process of platelet adhesion and aggregation. The PFA-200 P2Y_12_ allows detecting platelet ADP-receptor blockades using a membrane covered with 20 ug of ADP, 5 ng PGE1, and 125 ug of calcium. As recommended by the manufacturer we defined HPR as closure time (CT) <106 s (Siemens Healthcare. 2010. Guide insert. Innovance PFA P2Y.)

### 2.4. Adverse Reactions and Causality Analysis 

Patients were prospectively evaluated to establish the phenotypic response to treatment (bleeding or thrombosis). This evaluation was carried out through of periodic phone calls for six months and the completion of a monitoring form for each patient included in the study. At follow-up, the 13 patients who switched their antiplatelet therapy to ticagrelor or prasugrel were excluded.

Each time that an adverse event was aimed to obtain additional information from the patient’s medical records and data provided by the treating physician.

Each adverse event consisting of hemorrhage was subsequently evaluated in terms of its causality using the Naranjo algorithm [22]. The Naranjo algorithm was not used to evaluate therapeutic failures since this tool is not designed for this purpose. The Naranjo algorithm is a questionnaire proposed by Naranjo et al. for establishing the probability of whether an adverse drug reaction (ADR) was caused by the suspect drug (clopidogrel in our study) rather than concurrent factors (e.g., comorbidities). Probability was assigned using the following scores: definite, probable, possible, or doubtful. Every time the answer to any of the Naranjo algorithm questions was unknown, a score of “0” was allocated to the corresponding question, as it is established in the algorithm [22].

We defined the occurrence of major bleedings according to the criteria of Schulman et al. [23], these include: (1) fatal bleeding, and/or, (2) symptomatic bleeding in a critical area or organ, such as intracranial, intraspinal, intraocular, retroperitoneal, intra-articular or pericardial, or intramuscular with compartment syndrome, and/or, (3) bleeding causing a fall in hemoglobin level of 20 g L^−1^ (1.24 mmol L^−1^) or more, or leading to transfusion of two or more units of whole blood or red blood cells [23].

Two independent researchers evaluated the cases separately, and any disagreements were solved by consensus with a third author (pharmacologist).

### 2.5. Statistical Analysis

Allele and genotype frequencies and Hardy–Weinberg equilibrium were determined using the SNP-Stats software (https://www.snpstats.net/start.htm (accessed on 14 March 2021)). Deviation from HWE was estimated using a χ2 goodness-of-fit test with 1° of freedom. The χ2 test was used to compare allele frequencies between the Colombian population and others (a *p*-value of <0.05 was considered statistically significant). Linkage disequilibrium (LD) between intronic SNVs was determined by applying the LOD and D’ value on Haploview v4.2 (https://www.broadinstitute.org/ (accessed on 14 March 2021)). The groups were defined as Non-HPR or HPR based on the CT value obtained using the INNOVANCE PFA-200 P2Y system (Siemens Healthcare). The χ2 test was used to compare genotype and Non-HPR or HPR condition. Genotypes were categorized into two subgroups: extensive metabolizers (EM, including the diplotypes wild type and diplotypes with at least one allele*17) and intermediate metabolizers (IM, including diplotypes with at least one LOF allele). To compare CT value with EM or IM status, data distribution was evaluated using the Shapiro–Wilk test. Non-normally distributed data was found (*p* < 0.05) and nonparametric Mann–Whitney U test was used to compare groups.

## 3. Results

### 3.1. Clinical and Demographic Characteristics

The patients’ characteristics are summarized in Table 1. The majority were males (63.8%). Most cases were over 50 years old, and the predominant type of coronary syndrome was acute myocardial infarction with ST elevation (63.2%). 32.5% of patients previously presented one or more ACS when recruited into the study and nearly 30% presented a comorbidity (e.g., diabetes, obesity, or dyslipidemia) (Table 1). 

### 3.2. Analysis of Genetic Variants in CYP2C19

**Figure 1:**
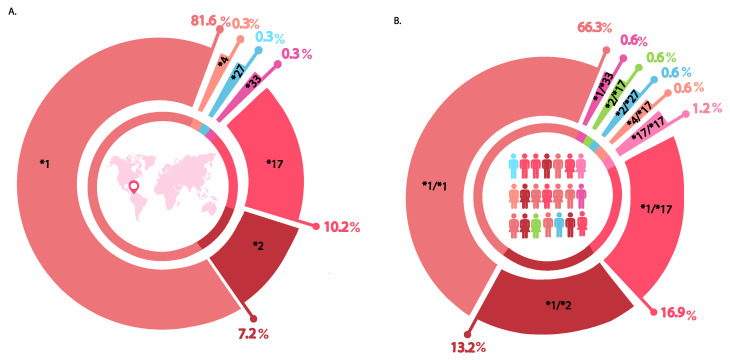
Allele and genotype frequencies. Data for allele and genotype frequencies of *CYP2C19* are illustrated. (**A**) Allele frequency and (**B**) genotype frequency.

*CYP2C19* was sequenced in the 166 ACS patients, and we identified a total of 41 single nucleotide variations (SNVs). For the analyzed population, the following allelic frequencies were established: 81.6% CYP2C19*1, 10.2% CYP2C19*17, 7.2% CYP2C19*2, and 0.3% for the *27, *4, and *33 *CYP2C19* alleles. (Figure 1A). 18.3% of the population were carriers of alleles that have been related to interindividual variability in response to clopidogrel. Furthermore, 66.3% of the identified genotypes were wild type, CYP2C19*1/*1 and 33.7% had a genotype with at least one polymorphic allele. From the last, 18.1% were ultrarapid metabolizers (*1/*17, *17/*17), 14.4% intermediate metabolizers (*1/*2, *4/*17, *2/*17) and 0.6% to slow metabolizers (*2/*27). There were no patients identified as homozygous for allele*2. One patient was identified as heterozygous for allele*33 (0.6%), whose metabolizer phenotype has not been established. (Figure 1B)

Two novel non-synonymous CYP2C19 variants were identified, both with an allele frequency of 0.3%. These variants correspond to c.44T>A p.L15H and c.1215G>C p.E405D. In silico analysis (SIFT, MutPred, PolyPhen-2) predicted the pathogenic effect of p.L15H, while p.E405D was predicted as benign. Alignment with different species showed evolutionary conservation for these two variants (Supplementary Figure S1).

**Figure 2:**
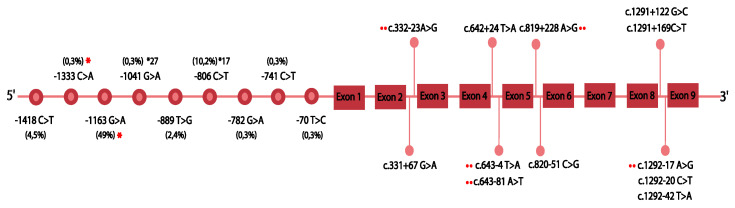
Promoter and intronic variants. *CYP2C19* genetic variants in promoter and intronic regions are described. Numbers in parentheses represent allele frequencies; * indicates novel variants and **¨** illustrates a predicted potential splicing alteration.

Twelve intronic variants were identified, in silico analysis predicted a potential splicing alteration for five of them. For c.332-23A>G, c.1292-17A>G and c.634-4 T>A, a branch point modification was identified. The software predicted activation of a cryptic acceptor and donor site for the variants c.643-81 A>T and c.819 + 228 A>G, respectively (Figure 2). Nine variants were identified in the promoter region; these correspond to −1418C>T, −1333C>A, −1163G>A, −1041G>A (*27), −889T>G, −806C>T (*17), −782G>A, −741C>T and −70T>C. From these variants, seven have been reported previously in the literature, while two are novel (−1333C>A, −1163G>A). Only two polymorphisms identified (−1041G>A and −806C>T) have been related to modifications in the CYP2C19 enzyme activity (Figure 2).

**Figure 3:**
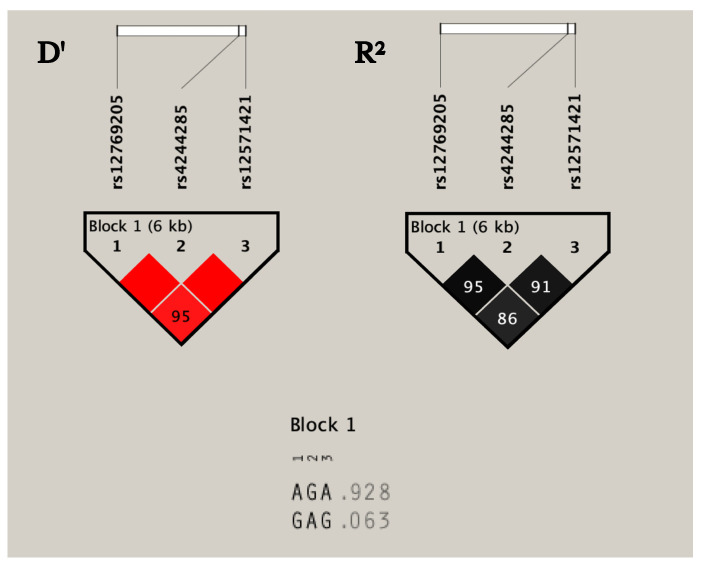
Linkage disequilibrium analysis. Linkage disequilibrium among the intronic single nucleotide variations (SNVs) and haplotype block analyzed by Haploview 4.2 software. The D’ value is shown within the square (D’ = 1, not shown). The three SNVs constitute one haplotype block spanning 6 kb of the *CYP2C19* gene. R^2^ value indicates the high correlation coefficients between SNVs.

LD analysis indicates that rs12769205, rs424428 and rs12571421 exhibit extremely significant linkage disequilibrium (LOD > 2, D’1, R2 ≥ 0.6). The three SNVs were all located in one LD block of 6kb. Haplotype analysis identified two haplotypes: AGA (92.8%) and GAG (6.3%) (Figure 3).

The comparison of allelic frequencies between the Colombian population and other populations showed statistically significant differences between the wild-type and polymorphic alleles (Supplementary Table S2). The Asian, Caucasian, African and Latin America (e.g., Argentina, Brazil) populations have a lower frequency for CYP2C19*1 compared to our study (*p* < 0.00001). Conversely, Ecuador México and Peru had CYP2C19*1 frequencies like that described by us (*p* = 0.91). Regarding LOF alleles for *CYP2C19*, our findings demonstrated that allele frequency for CYP2C19*2 in Colombian patients was significantly lower than that reported for other populations. Strikingly, allele frequency for Asian populations is 0.28 whereas for our population is just 0.7 (*p*-value < 0.00001). No significant differences were detected with other Latin American populations (Argentina, Brazil, Ecuador, Mexico). CYP2C19*4 allelic frequency was low in our study (0.3%), consistently with studies in other populations. Other LOF alleles identified in our study (*27) have not been extensively evaluated in other reports. Regarding CYP2C19*17 allelic frequencies, our data showed differences with most Latin American, Asian, and African populations, where CYP2C19*17 is more frequent (Supplementary Table S2).

### 3.3. Reactivity Platelet Function and Genotype

The mean closure time (CT) values assessed using the INNOVANCE PFA-200 P2Y system (Siemens Healthcare) were 210 +/−110 s (range 37–300). 34% patients showed a CT value suggestive of HPR. Due to the low frequency of polymorphic *CYP2C19* alleles, carriers of at least one LOF variant (*2, *4, *27) and UR variant (*17) were compared with wild-type individuals. The non-HPR and HPR group had not a statistically significant difference between genotype frequencies (Table 2). Corresponding p-values after comparison were *p* = 0.21 for loss-of-function and *p* = 0.83 for gain-of-function.

Although we did not find a statistically significant difference in ranges between CT vs. EM or IM (*p* = 0.315), the range in EM subgroups was higher than those of IM patients (84.82 and 74.86, respectively).

### 3.4. Clinical Adverse Reactions and Causality Analysis

We identified 23 patients with adverse events. Considering that some patients had more than one adverse event the total number was 28.

Seven of these events were fatal, but only one of them was related to bleeding/thrombosis. Three therapeutic failures occurred (one case of ischemic stroke and death and two of acute myocardial infarction). Both patients with acute myocardial infarction had stent thrombosis. One of them had a stent in the right coronary artery and in the second case the clinical history indicates the use of a stent in the anterior and posterior descending coronary artery. 19 minor bleedings were considered adverse reactions to clopidogrel (Table 3). No major bleedings were documented in the study according to pre-established criteria. Regarding causality, without considering therapeutic failures, 44% of the ADRs were classified as possible and 32% were probable. 

## 4. Discussion 

Clopidogrel therapy has shown different clinical outcomes that depend on the efficacy of the drug. HPR has been linked to major adverse cardiovascular events (MACE) and LPR with bleeding, both conditions have a great clinical impact and represent a challenge for the safe and effective use of the drug [16]. Clopidogrel is a second-generation thienopyridine widely used to treat the acute coronary syndrome. It is administered as an inactive prodrug that requires hepatic conversion by cytochrome P450 (CYP) enzymes, mainly 2C19 to produce an active metabolite [6]. *CYP2C19* polymorphisms are related to the anti-platelet effects of clopidogrel [4,7,13,24]. To the best of our knowledge, this study is the first to assess the variants of the promoter region, the intronic and coding region of *CYP2C19* through sequencing in a group of Colombian patients with ACS and determines the association with platelet reactivity. In this study, we identified 41 genetic variants, including new polymorphisms in the coding and regulatory *CY2C19* regions. 

Allele frequencies of *CYP2C19* *1 were up to 1.4 times higher than those assessed in other populations (Caucasian, Asian, African, and Latin American) (*p* < 0.05). Interestingly, CYP2C19*17 has a lower allele frequency (2.1 times) concerning populations of other ethnic origins, including Latin America [2,25]. The main LOF CYP2C19*2 allele, previously related to interindividual variability of response to clopidogrel behaves similarly between Latin American populations but presents significant differences with Asian and European populations, in which this allele reaches values of 28% [26]. These results suggest that the Colombian population presents a pharmacogenetic profile of less susceptibility to low and high platelet reactivity in patients treated with the antiplatelet clopidogrel compared to other Latin American or world populations. Recently the RIBEF (Consortium of the Ibero-American Network of Pharmacogenetics and Pharmacogenomics) analyzed the frequency of *CYP2C19* variants in several Latin American countries, nevertheless, Colombia was not included [25,27]. The results of this study contribute to fill this knowledge gap in the Latin American populations underlining high degree of genetic admixture. Colombia is the country with the highest inter-population variability, described as nearly 49% being of mixed origin, 37% of European origin, 10% of African origin, and 3.4% of Amerindian origin [23]. 

Regarding the population impact of pharmacogenetic relevant alleles in response to clopidogrel, 33.7% of patients analyzed were carriers of at least one variant in *CYP2C19* related to gain or loss of function (10.2% and 7.8% respectively). These findings allow us to infer that similar to other Latin American countries, the impact of LOF variants is much lower compared to Asian populations where the frequency of these variants is five times higher [26]. The low frequency of CYP2C19*17 revealed that our population is potentially less susceptible to developing low platelet reactivity during treatment with clopidogrel in relation to Caucasian or African populations, where the frequency of CYP2C19*17 is 21% and 17%, respectively. These findings emphasize the importance of using population genetic approaches to determine the real impact of specific populations’ drug responses. 

Loss-of-function alleles represented 7.8% of the total and from these, CYP2C19*2 represented 7.2% (Figure 1). This allele is most frequently associated with high residual platelet reactivity (HPR) in clopidogrel therapy. HPR is a relevant marker in adverse cardiovascular outcomes and has been used to individualize antiplatelet therapy [28]. Clinical relevance of CYP2C19*2 has been described as well as association with risk of cardiovascular events, stroke and major bleeding [29,30]. These findings have justified the implementation of clinical management guidelines based on genotype, which suggest that new P2Y_12_ inhibitors such as prasugrel and ticagrelor are alternatives for patients who are carriers of LOF genetic variants for *CYP2C19.* Screening for the presence of loss-of-function alleles could allow more informed decision-making regarding the choice of the P2Y receptor blocker [15,24,31].

The frequency for the gain-of-function allele CYP2C19*17 was 10.2%. This allele has been widely characterized and related to ultrarapid metabolism of *CYP2C19*. CYP2C19*17 has been associated to low risk of cardiovascular events, high risk of major bleeding, and lower PR on-treatment [32]. These outcomes are attributed to the fact that patients with the allele CYP2C19*17 are proportionally more exposed to the active metabolite of clopidogrel, which results in a higher inhibition of platelet aggregation to wild type individuals [33]. According to the results of our study, we can hypothesize that patients under treatment with clopidogrel from our population will have a lower risk of suffering major adverse cardiovascular events and bleeding have a greater impact in antiplatelet treatment. Genotypes related to ultra-rapid or extensive phenotypes (18.1%) were more prevalent respect to the poor (0.6%) or intermediate metabolism (14.4%). CYP2C19*17 is defined by an SNV located in the promoter region (c.−806C>T), functional validation using electrophoretic mobility shift assays (EMSA) revealed binding of hepatic nuclear proteins at −806T but not at −806C. Increased transcriptional activity of -806T allele was confirmed in murine models [34]. 

Interestingly, analysis through reporter genes has revealed a remarkable heterogeneity in the luciferase activity in CYP2C19 *1/*1 individuals, attributed to non-identified SNV in the *CYP2C19* 5’upstream region [34]. Our study led to the identification of 9 SNVs in the −1500 pb promoter region, two of which correspond to novel variants (−1333C>A, −1163 G>A). 0.6% of the patients were carriers of the allele CYP2C19*27 (c.−1041G>A), for which a significantly reduced luciferase activity in transfected constructs in HepG2 cells has been reported [35]. In silico analyses have suggested that this effect is potentially related to the generation of a new repressor transcription factor binding site with the OCT-1 [35]. Interestingly, the SNV −70T>C, identified in 0.6% of patients, is located in a positive regulatory region of *CYP2C19.* Arefayene et al., conducted a functional study using artificially generated nested deletions in the gene promoter and studied the ability of allele series to activate luciferase expression in the HepG2 cell line. The data indicated that deletions in CYP*2C19* c- −17 to −153 pb generate a significantly reduced activity. This finding was according with potential alteration of factor transcription binding. [36].

The diversity of SNVs identified in the promoter region of *CYP2C19* [36,37,38], permit us to infer that genetic variants in this region might contribute to interindividual variation in enzymatic activity in vivo. Functional validation of these SNVs will light in the understanding of the expression and heterogeneous activity of *CYP2C19*. Additionally, future studies are necessary for improved understanding of CYP2C19 expression, heterogeneous activity and the unexplained interindividual variability response to clopidogrel of CYP2C19 *1/1 subject. High allele-frequency variants (e.g., −889 T>G) are of particular interest due to the magnitude of their potential effect at population level.

Regarding intronic variants, the SNP c.332-23A>G (CYP2C19*35) co-segregated with c.681 G>A (CYP2C19*2) was identified in 24 patients. Previous studies have indicated a complete linkage disequilibrium between these two markers (LOD>2, D’ = 1) [39]. Additionally, we show that the polymorphism c.819 + 228 A>G is potentially related with a splicing alteration, activation of a cryptic donor site, and was part of the haplotype block formed by the SNVs c.681 G>A (CYP2C19*2) and c. 332-23 A>G (CYP2C19*35) which indicates a high LD among the three variants (Figure 2). This finding has not been previously reported and is relevant considering the potential functional impact of this co-segregation due to the generation of multiple aberrant transcripts with drastic effects on the protein. Previous reports have shown that the haplotype involving the*2 and*35 variants generate transcripts with total retention of intron 2 (exon 2B), a 40 bp deletion of exon 5 and a 71 bp deletion of exon 4 that creates a premature stop codon in exon 4 [39]. The presence of a third variant with a potential effect on splicing could lead to the generation of additional aberrant transcripts.

The in silico analysis of the intronic SNVs c.1292-17 A>G, c.634-4 T>A and 332-23 A>G (*35) suggest a potential alteration in the branch point site (BPS). These variants generate or eliminate the adenine that is part of a key conserved sequence, critical for the assembly of the spliceosome and lariat intron formation, changes in BPS can result in aberrant pre-mRNA splicing [40]. As previously mentioned, c.332-23 A>G (*35) generates a functional effect in *CYP2C19*, which allows us to hypothesize that new intronic SNVs can generate alleles that affect the protein [39]. Functional validation (e.g., minigenes) will allow to assess the biological effect of haplotypes and intronic variants, which given their molecular involvement would correspond to possible LOF alleles for *CYP2C19*.

Recently, Morales-Rosado et al. conducted a NGS (next generation sequencing) analysis in *CYP2C19*, and reported potentially disruptive intronic alleles in 16% of patients [41]. Similarly to our study, they were located towards the end of the gene. We show that 41.6% of the polymorphisms were found in intron 8 (Figure 2). Usually, these types of variants are not included in pharmacogenetic analyzes but due to their role in the regulation of alternative splicing and gene regulation, they constitute new potential response factors to the interindividual variability of the response to clopidogrel [41].

This study identified two novel exonic variants, c.44T>A (p.L15H) and c.1215G>C (p.E405D). *CYP2C19*-p.L15H was identified in the patient SCA_136; in silico analysis suggested its pathogenicity due to amino acid conservation during mammalian evolution, which might be related to a role in its biological function. Indeed, *CYP2C19* is inserted co-translationally in the membrane of the endoplasmic reticulum using signals directed by the N-terminal hydrophobic domain of the protein [42,43]. Functional expression and cellular localization studies of plasmids with mutant proteins for the N-terminal (amino acids 3 to 20) showed that changes in this region modify the degree of integration, potentially affecting subcellular localization and enzyme activity [42]. Variants located in N-terminal end such as p.L16F leads to decreased expression level [44]. *CYP2C19*-p.405E is a residue located in the L Helix, implicated in the binding of the heme group [45]. Functional analysis has shown that genetic variants in *CYP2C19* that modify residues located in or near the binding region of the heme group can alter the stability and/or efficiency of the folding of the holoprotein CYP2C19, which leads to the potential degradation of functional proteins [46]. Despite the conservation of E405 residue and, in silico analysis, CYP2C19-p.E405D could have a functional effect that needs to be validated.

Our study determined platelet function using the INNOVANCE PFA-200 system (Siemens Healthcare, Germany) which assesses P2Y12-receptor blockades in patients undergoing therapy with P2Y12-receptor antagonists [47]. Despite some reports indicating a positive correlation between the CT value, HTPR, and MACE frequency [13,20,48,49], our findings failed to associate the molecular genotype and phenotype of HPR. This result can be attributed to several factors; (a) low frequency of LOF alleles (b) absence of patients with the homozygous genotype for LOF alleles (c) high frequency CYP2C19*1/*1 with CT suggestive of HPR (d) potential overestimation of patients HPR (technical limitation of PFA-200); (e) involvement of new intronic and/or promoter variants and (f) heterogeneity of response to clopidogrel due to genes not evaluated in this study [5,50].

Similarly, it has been established that the use of platelet function tests such as PFA-200, Verify Now P2Y_12_ assay and others are potentially predictive of the clinical response to clopidogrel [20,49]. Although not significant, intermediate metabolizers (at least one loss function allele) showed a trend towards decreasing CT values and therefore are more susceptible to HPR. Possibly the absence of poor metabolizers and the low proportion of carriers in our population are responsible for the observed non-significance. Studies using the PFA-200 platelet function test in Asian population, where 59% of patients had *CYP2C19*-loss-of-function genotypes, found that CT values were significantly different between EM and IM or PM suggesting a significant association between the efficacy of clopidogrel and *CYP2C19* genotypes [20]

The clinical outcome could be evaluated in 97% of the subjects included in the study (with continuous clopidogrel therapy), 24 patients with adverse reactions were identified, with a predominance of bleeding (Table 3). The occurrence of these events is explained mainly at the pathophysiological level by low or high platelet reactivity, attributed in part to alleles related to UR or slow metabolism for *CYP2C19*. The results of the causality analysis evaluated by the Naranjo algorithm estimated that clopidogrel was the probable or possible cause of 76% of the adverse reactions observed in the patients. Despite not reaching statistical significance between polymorphic alleles and platelet reactivity, we observed that 25% of patients with ADR were carriers of genetic variants of known functional impact (*2, *17). The existence of polymorphisms in regulatory regions (introns and promoter) could explain the heterogeneity in the clinical response to clopidogrel observed by us [41]. In addition, if it is considered that the interindividual variability to antiplatelet treatment attributed to CYP2C19 polymorphisms is 12%, variants in other genes not analyzed could influence the appearance of ADR in people with the CYP2C19*1/*1 genotype [4].

In addition to the potential genetic impact on the generation of adverse drug reactions (ADRs) to clopidogrel, the Colombian clinical practice guidelines for acute coronary syndrome (ACS) recommend a lower loading dose that can impact the presentation of ADRs. In this precise manner, the loading dose of 300 mg recommended at the time of hospital admission is lower as compared to the European society of cardiology guidelines (600 mg) [51].

Given the clinical evidence that has found a higher percentage of bleeding with the 600 mg vs. 300 mg dosage of clopidogrel (1·6% vs. 1·1%, *p* = 0·009) [52], it is plausible to think that our patients can present this adverse reaction lower than expected. Even so, most of our ARDS correspond to bleeding; this evidence supports that other factors (ej. UR metabolism allele carrier *17) can influence the presentation of unwanted effects.

The use of 600 mg loading dose for clopidogrel recommended by the European guidelines was justified through the evidence collected mainly on the CURRENT-OASIS 7 trial, which demonstrated a reduction in major cardiovascular events and stent thrombosis as compared to the lower 300 mg loading dose [53]. Our national guidelines take this evidence into consideration and recommend giving 300 mg to every patient with ACS, and an additional 300 mg (completing 600 mg of loading dose) when the patient can be guaranteed an early percutaneous coronary intervention (PCI). In our study, a therapeutic failure was found in the setting of an Acute myocardial infarct secondary to stent thrombosis, this patient did not receive the 600 mg loading dose of clopidogrel. This case evokes the fact that lower doses can be a potential cause for the MACEs previously reported [53,54]. Taking into account the genetic implication existing in our population with a lesser frequency of alleles related to high platelet reaction that potentially favors the lower presentation of MACEs despite the lower dosages recommended for the country.

Regarding the use of other antiplatelet agents such as ticagrelor and prasugrel, despite the evidence found, in terms of effectiveness and security, they are not first-line medications in our country, and they require additional logistic clearance for their use since they are contemplated outside of the national mandatory plan (https://tablas.sispro.gov.co/TestMiPresNopbs/ModTest/Mipres.aspx (accessed on 14 March 2021)). Furthermore, these agents can generate over costs in countries with budget constraints [53]. These observations can explain the low proportion of patients in whom a change of use of Clopidogrel to ticagrelor or prasugrel was seen.

Taken together, our results allowed us to establish the pharmacogenetic profile of *CYP2C19* in Colombian patients with ACS under clopidogrel treatment. Importantly, differences in allele frequencies responsible for the interindividual variability attributed to the safe and effective antiplatelet response were identified in our population. Additionally, *CYP2C19* sequencing identifies intronic variants potentially related with splicing modification, which are not currently covered by pharmacogenetic analysis of clopidogrel therapy. The association of some of these SNVs and clopidogrel pharmacokinetics needs to be further validated.

Our study has several limitations. The low frequency of alleles related to slow and ultra-rapid metabolism together with the absence of homozygous for LOF alleles of CY2C19 limits the power to detect a significant association between molecular genotype and platelet reactivity. Functional validation is required to determine the biological significance of deep intronic variants and promoter region polymorphisms. However, the identification of polymorphisms that are not usually part of the pharmacogenetic analysis of *CYP2C19* is relevant for the identification of new alleles. Finally, clinical follow-up for a six-months period may be insufficient for the detection of major cardiovascular events.

## 5. Conclusions

In conclusion, the present study allowed us to identify *CYP2C19* variants in patients affected with ACS and treated with clopidogrel. Allele frequencies for some of the polymorphisms identified in the Colombian population were statistically different to those in other populations and globally, which suggests a specific susceptibility. Given our findings regarding allele frequencies of loss or gain of function, we can infer a greater effect of low platelet reactivity in patients treated with clopidogrel. Furthermore, in silico analysis for new variants in the coding and regulatory regions allowed us to propose new molecular mechanisms potentially related to interindividual variability in response to clopidogrel. Ultimately, our results contribute to the understanding of the population pharmacogenetics in Latin America.

## Figures and Tables

**Table 1:** Patients’ characteristics.

Characteristics	(n)	%
Sex	166	100
Female	60	36.1
Male	106	63.8
Age (years)		
30–50	11	6.6
50–70	88	53
>70	67	40.3
Type of ACS		
UA	25	15
STEMI	105	63.2
NSTEMI	36	21.6
Previous ACS medical history *	54	32.5
Type of intervention current event		
Medical ^†^	44	26.5
PCI	86	51.8
CABG	36	21.6
Stent placement current event	73	43.9
Type 2 Diabetes Mellitus		
Body Mass Index	46	27.7
Underweight	2	1.2
Normal	58	34.9
Overweight	64	38.5
Obese	42	25.3

ACS, acute coronary syndrome; UA, unstable angina; STEMI, ST elevation myocardial infarction; NSTEMI, non-ST myocardial infarction; PCI, percutaneous coronary intervention; CABG, coronary artery bypass grafting. * Patients who had previously presented one or more ACS when recruited into the study. ^†^ Medical Management, supportive and pharmacologic care.

**Table 2:** Non-high platelet reactivity (HPR) and HPR statistical comparison.

Genetic Variants Genotypes	All	HPR	Non-HPR	
	n (%)	n (%)	n (%)	*p*-Value
*CYP2C19* Loss function alleles				
Wild type	143 (86)	46 (27.7)	97 (58.4)	0.21
Carrier	23 (14)	11 (6.6)	12 (7.2)	
*CYP2C19* Gain function alleles				
Wild type	134 (80.7)	45 (27.1)	89 (53.6)	0.83
Carrier	32 (19.2)	12 (7.2)	20 (12)	

*CYP2C19* loss-function-alleles includes *2, *4, *27, *33. *CYP2C19* gain-function-alleles correspond to *17. Values are represented in n (%). Abbreviations: HPR, high platelet reactivity.

**Table 3:** Clinical adverse reactions and causality analysis.

**Therapeutic failures**	3
Acute myocardial infarction	2
Ischemic stroke and death	1
**Bleedings (all minor)**	19
Ecchymosis	7
Epistaxis	4
Gingivorrhagia	2
Hematemesis	1
Petechiae	1
Rectorrhagia	1
Spontaneous bruising	2
Uterine hemorrhage	1
**Causality (ADR excluding FT cases)**	
Probable	8
Possible	11
Unclassifiable	(fatal cases)

Causality was evaluated using the Naranjo algorithm. Abbreviations: ADR: adverse drugs reactions, FT; fatal cases.

## Data Availability

The data presented in this study are available on request from the corresponding author. The data are not publicly available as they correspond to Sanger sequencing and closure times values.

## Supplementary Materials

The following are available online at https://www.mdpi.com/article/10.3390/jpm11050400/s1, Figure S1: Comparison between sequences of proteins with modified residues and orthologous proteins of mammalian species. Table S1: Primers sequence. Table S2: The comparison of allelic frequencies between the Colombian population and other populations.
