# Supplementary material for: A Pharmacogenetic Study of CYP2C19 in Acute Coronary Syndrome Patients of Colombian Origin Reveals New Polymorphisms Potentially Related to Clopidogrel Therapy
Source: J Pers Med. 2021 May 12;11(5):400. doi: 10.3390/jpm11050400 (PMC8150782; doi:10.3390/jpm11050400)
Supplement: Supplementary file 1 [file jpm-11-00400-s001.zip › Supplementary Figure 1.pdf]

		10	11	12	13	14	15	16	17	18	19	20	21	22
P33261	CP2CJ_HUMAN	C	L	S	C	L	L	L	L	S	I	W	R	Q
A0A2I3RMB5	A0A2I3RMB5_PANTR	C	L	S	C	L	L	L	L	S	I	W	R	Q
G3QCV6	G3QCV6_GORGO	C	L	S	C	L	L	L	L	S	I	W	R	Q
G1RRD4	G1RRD4_NOMLE	C	L	S	C	L	L	L	L	S	L	C	R	Q
A0A2R9B7Q9	A0A2R9B7Q9_PANPA	C	L	S	C	L	L	L	L	S	I	W	R	Q

********** **

c.44T > A - p.L15H

Prediction pathogenic score

SIFT = 0 ; Damaging

MutPred = 0. 564 ; Pathogenic

PolyPhen-2 = 0.999 ; Probably Damaging

		399	400	401	402	403	404	405	406	407	408	409
P33261	CP2CJ_HUMAN	K	E	F	P	N	P	E	M	F	D	P
A0A2I3RMB5	A0A2I3RMB5_PANTR	K	E	F	P	N	P	E	M	F	D	P
G3QCV6	G3QCV6_GORGO	K	E	F	P	N	P	E	M	F	D	P
G1RRD4	G1RRD4_NOMLE	K	E	F	P	N	P	E	M	F	D	P
A0A2R9B7Q9	A0A2R9B7Q9_PANPA	K	E	F	P	N	P	E	M	F	D	P

****

c.1215G > C - p.E405D

Prediction pathogenic score

SIFT = 0.169 ; Tolerated

MutPred = 0.425 ; Benign

PolyPhen-2 = 0.001 ; Bening
